# An ethnographic discourse approach to explore GP-counsellor communication in primary health care settings in Hong Kong

**DOI:** 10.3389/fpsyg.2022.943840

**Published:** 2022-12-12

**Authors:** Jack Pun, Qianwen Joyce Yu, Matthew K. C. Lee

**Affiliations:** Department of English, City University of Hong Kong, Kowloon, Hong Kong SAR, China

**Keywords:** GP, counsellor, primary care, Hong Kong, ethnographic discourse analysis

## Abstract

This study aims to explore the nature of GP-counsellor interaction during discussions of patients’ mental health issues in primary care services. An ethnographic discourse analysis of audio-recorded GP-counsellor conversations and the participating counsellor’s reflective accounts. Two participating GPs and one counsellor were recruited from a private medical clinic in Hong Kong. The GPs and the counsellor mainly discussed their patients’ issues in terms of medication management, the counsellor’s case conceptualization, the case management, knowledge transfer and acknowledging the partnership. During case discussions, both the GPs and the counsellor used a range of interactional strategies to clarify a patient’s condition and treatment plans for mutual understanding. The GPs and the counsellor co-construct an insider discourse that covers a greater diversity of topics, including both medical and non-medical concerns. The research findings have implications for theory and practice, including the potential of ethnographic discourse analysis in understanding the features of participants’ behavior and evaluating the effectiveness of communication in a particular setting, as well as the importance of exploring professionalized discourse during GP-counsellor communication in developing training programs aimed at enhancing staff awareness of effective IPC communication.

## Introduction

Interprofessional collaboration (IPC) has nowadays been recognized as an essential ingredient for patient-centered care, functioning as a response to growing chronic disease burden, a rapidly aging population, the sophisticated therapeutic modalities, and the escalating healthcare costs (Fox and Reeves, 2014; Reeves et al., 2017). An interprofessional team-based approach has been increasingly prioritized in primary care contexts (Mulvale et al., 2016), with a particular view to addressing the medical needs of those suffering from chronic diseases (Lee et al., 2014; Careau et al., 2015). However, the primary contexts can be challenging for IPC. Reasons include the nascent development of IPC in primary care practice compared to other healthcare contexts (Fox et al., 2021), the wide scope of clinical work in primary care (Delva et al., 2008), and the deeply entrenched medical hierarchy (Abbott, 2014; Albassam et al., 2020) characterizing work interprofessional relationships in primary care. A deep understanding of IPC in primary care is thus of critical importance.

Communication is a key element to effective IPC (San Martín-Rodríguez et al., 2005). Yet little research on IPC has been performed in investigating the actual communicative practices involved (Denvir and Brewer, 2015; Fox et al., 2021). To address the gap, this empirical paper focuses on a frequent and potentially difficult type of IPC in primary care: communication between general practitioners (GPs) and counsellors. By doing so, we seek to demonstrate the nature of GP-counsellor interaction during discussions of patients’ mental health issues in a Hong Kong primary care setting.

## Background

### Mental health-primary care collaborations

IPC is crucial to strengthening healthcare systems, improving patient outcomes, increasing patient and provider satisfaction, and delivering patient-centered healthcare services (World Health Organization, 2010). In primary care, how IPC works is largely shaped by the multifaceted nature of first-line treatment (Fox et al., 2021), which interwoven with different demands and tasks, including medical care, health promotion, social care, and disease prevention activities (Xyrichis and Lowton, 2008). An interprofessional team approach in primary care is especially useful in treating patients with complex and chronic conditions such as mental disorders (Pullon et al., 2016).

As major contributors to worldwide disease burden, mental disorders are highly prevalent in primary care (World Health Organization and World Organization of Family Doctors, 2008). Since the declaration of Alma-Ata (World Health Organization, 1978), integrating mental health service into primary care has become the most feasible means to close the treatment gap (between the prevalence of mental illness and the number of patients receiving medical care and treatment), to ensure that individuals get the mental health care they need, and to facilitate patient-centered and holistic healthcare services (World Health Organization and World Organization of Family Doctors, 2008).

Existing research has repeatedly linked mental health-primary care collaborations to positive health-related effects. These include improved screening and detection of psychiatric conditions (Gilbody et al., 2006; Oslin et al., 2006), collocating mental healthcare services in primary care, presenting medical issues from mental and physical health perspectives (Blount, 2003; Sperry, 2013), reduced indirect costs related to seeking medical help in remote locations, minimized stigma and discrimination, removed risks of human rights violations occurring in psychiatric hospitals (World Health Organization and World Organization of Family Doctors, 2008).

### Communication between general practitioners and counsellors

Communication is a crucial determinant of effective IPC (San Martín-Rodríguez et al., 2005). Effective interprofessional communication facilitates constructive negotiations and functions as a vehicle for other IPC determinants such as mutual respect, sharing and trust, ultimately contributing to high outcomes (San Martín-Rodríguez et al., 2005; Weller et al., 2014; The Joint Commission, 2015). Ineffective communication, however, leads to delayed treatment, medication errors, misdiagnosis, poor patient safety and sentinel events (The Joint Commission, 2015).

Despite its significant role in IPC, communication between general practitioner (GP) and counsellor during mental health-primary care collaborations can be challenging. The two parties manage mental health cases from different perspectives due to different ideologies and modalities. The biomedical model assumes mental disorders derive from pathogenic-related brain disease and emphasizes biological treatment (Andreasen, 1985). Treatment is understood as the responsibility of medical professionals and its goal is to discover the therapeutic agents that target the disordered somatic processes without harming the organism (Moncrieff, 2008). For example, GPs tend to give prescriptions related to serotonin levels for patients with depressive symptoms and may not attribute the presenting problems or symptoms to the patients’ mental state or behavior. In contrast, counsellors are used to working on somatic complaints from a broader biopsychosocial perspective and their work relies heavily on the reciprocal relationship with the client (Gunn Jr and Blount, 2009; Foon et al., 2020; Wattanapisit et al., 2021). For instance, GPs may explain the cause of depression as an imbalance of neurotransmitters and medication is often recommended as treatment; whereas counsellors often explore various factors that may lead to the manifestation of mental disorders.

These challenges set the scene for a complex and demanding situation for both GPs and counsellors. Despite different understandings and accounts of mental disorders, both GPs and counsellors understand the complexities surround each case of mental health distress and recognize the importance of interdisciplinary help. However, little research on IPC has been performed in investigating the actual communicative practices involved (Denvir and Brewer, 2015; Fox et al., 2021) and empirical evidence is even scarcer when it comes to GP-counsellor communication. The few studies addressing GP-counsellor communication have tended to be quantitative, assessing the quality of IPC and exploring the factors related to satisfactory IPC (Vergès et al., 2020). While offering insights into stakeholders’ perspectives of communication, these studies fall short of examining how GP’s and counsellors’ communication strategies translate into actual IPC interaction. A focus on GP-counsellor communication can not only offer insights into what challenges and opportunities the two parties face in their collaboration and how they strive to improving the quality of IPC, but it can also generate useful information for GPs, counsellors, and other healthcare professionals whose work involves interprofessional collaboration. An analysis of the processes through which GPs and counsellors bring together and negotiate their knowledge and working methods can contribute to a better understanding of the complexities of mental health-primary care collaboration. To address the gap, this study aims to explore and identify the crux of the GP-counsellor interaction with a focus on the role of counsellor, and investigate the interactional strategies that GPs and counsellors use to negotiate and clarify their understandings of their patients’ mental health issues and treatment plans.

## Materials and methods

### Participants

Two participating GPs and one counsellor were recruited from a private medical clinic in Hong Kong, where interprofessional collaboration was an established way of working. A convenience sampling technique for was adopted for data collection. Given the long-term commitment according to the requirements of the research (i.e., the participants would allow for audio-recordings of their professional practice and engage in writing reflective accounts for 3 months), only the three participants consent to their participation. We believe that it is reasonable to conduct the study with three participants in order to obtain in-depth data on their communication. All participants had been working together since 2006. A multidisciplinary team involving experts in applied linguistics, health communication and counselling was involved.

All participants were given an information sheet at the clinic explaining the research purpose, and the rights and confidentiality of the participants. Written consent forms were obtained prior to participation. Personal identifiers were removed after data collection, and all data obtained remained confidential. The study was approved by the CLASS human research ethics committee of the City University of Hong Kong.

### Data collection

Upon receiving written consent, we placed an audio-recorder on the table inside the consultation room, and left the room to reduce the observer’s effect. Conversational data consisted of 10 audio-recorded conversations, totaling 84 min, between two GPs and one counsellor in a private clinic in Hong Kong. Audio recordings were transcribed into Cantonese and then translated into English. A bilingual researcher used the NVivo software package for content analysis to ensure the accuracy of the medical-related terms and expressions. A bilingual research assistant with extensive training in transcription and translation performed a final double-check against the original audio-recordings. In addition to audio-recordings, the counsellor was invited to write reflective logs based on his own experience of communicating with GPs. In the logs, the counsellor provided an account of the IPC circumstances, what he has achieved professionally during IPC, and his present feelings about collaborating with GPs in primary care.

### Data analysis

We applied an ethnographic discourse approach (Macgilchrist and Van Hout, 2011; Pun et al., 2017) to analyze the interaction transcripts and unpack the quality of interaction sequences. Using complementary methods derived from ethnography and discourse analysis, this approach combines ethnography and discourse analysis to account for how participants use language for meaning construction in order to achieve their institutional goals within a particular social context (Pun, 2020). The data transcripts were mapped along three levels on three levels: (a) structural mapping (i.e., counting the verbal contributions in discussions between the GPs and the counsellor); (b) thematic mapping (i.e., identifying the recurring themes discussed by the GPs and the counsellor) and (c) discourse mapping (i.e., the interactional strategies used by the GPs and the counsellor for clarifying their understandings regarding patients’ conditions; Sarangi, 2010). We explored the ways that the counsellor initiated, responded and followed-up regarding the GPs’ explanations of each patient’s case, and then identified discourse strategies (i.e., openness or closedness, question types, turn-taking, pausing) that the counsellor used for clarifying information. We also investigate the interpersonal aspects of Cantonese expressions, such as how the counsellor asks questions, raises concerns, and seeks clarifications from the GPs. The current observational study follows the Strengthening the Reporting of Observational Studies in Epidemiology (STROBE) protocol (von Elm et al., 2007).

To quantify these data and to code the observed patterns computationally, a coding framework designed for translational research (Pun et al., 2017) was used to capture the sequence of communicative practices. The flow of communication surrounding GPs’ explanations of each case and related medical conditions at different stages of medical care were tracked (e.g., patient history, diagnosis-making and translations of medical information in a bilingual environment). The analysis generated more linguistic evidence (e.g., speech functions) on how the counsellor and the GPs exchanged information about the patients, and the ways that both parties construed their experiences and expertise. To check the reliability of the coding framework used in this project, approximately 10% of each type of data was independently coded by two raters, according to the coding sheet. An inter-rater reliability of (κ > 0.8) was achieved.

The analysis generated understanding of the key types of information being exchanged, as well as of the ways they interacted in transferring cases and shaping the decision-making process. In this regard, we could also understand the participants’ reflections on communicative practices as we sought to uncover the hidden messages in the conversations.

For analysis of the counsellor’s reflection logs, a thematic analysis was conducted using grounded theory to capture re-occurring topics mentioned by the GPs and the counsellor concerning their views on their communications. We first read through the transcripts carefully and gave initial free codes to segments relevant to communication. Several rounds of comparing, sorting, and re-coding were then carried out to identify connections among the coded segments. In this way, major themes were identified that related to factors potentially affecting GP-counsellor communication.

## Findings

The analysis of GP-counsellor interaction is presented under three major features: (a) structural mapping: the amount of talking by each participant; (b) discourse mapping: the interactional strategies they used to negotiate and clarify their understandings of patients’ mental health issues and treatment plans; and (c) thematic mapping: the recurring topics in conversations.

### Amount of talking in GP-counsellor interaction

To paint a picture of actual GP-counsellor communication, audio-recordings were converted into transcripts and the numbers of utterances (in Chinese characters) were counted, showing how much both parties contributed to discussions. Table 1 shows the amount of total talk: (a) overall; (b) regarding new case referrals and (c) regarding follow-ups. In most cases, the GPs’ amount of talk was nearly double that of the counsellor. Among these discussions, five concerned new case referrals and five concerned follow-up cases. A typical new case referral meeting involves a GP providing the counsellor with a significant amount of information about the patient to facilitate referral; while a case follow-up meeting involves the GP and the counsellor exchanging observations and insights about the patient they are seeing.

**Table 1:** The amounts of talk in the GP–counsellor interactions.

	The amounts of total talk (nos. of words)		
	Overall (*n* = 10)	New case referrals (*n* = 5)	Follow-ups (*n* = 5)
GPs	12,024	5,473	4,153
Counsellors	6,575	1,037	5,538

In new case referrals, the GPs talked four times more than the counsellor (see Table 1), suggesting that the GPs offer a significant amount of information about the new cases. It also implies that the GPs regarded such information as critical and that the counsellor should know it prior to meeting the new patient. Typical and recurrent topics included the patients’ previous clinical encounters, presenting problems, expectations, medication management and other relevant details. These reports were further analyzed and categorized using a systemic approach, the details of which are provided in the following section.

Proportional amounts of talk were found in discussions of follow-up cases (see Table 1), with the counsellor talking more. This pattern implies that the counsellor contributed more to the talks after starting counselling sessions with the patients.

### Interactional strategies between GPs and the counsellor

Drawing from the coding framework (Pun et al., 2017), we identified the speech functions in these conversations to reveal how the case meetings developed in terms of the speech roles performed by the GPs and the counsellor. Possible categories of how the counsellor and GPs engage in communication were identified, including statements, closed-ended questions, open-ended questions, acknowledgements, and verbal acts of checking, confirming, clarifying, disengaging, challenging, and countering.

Table 2 presents a summary of the identified speech functions. The counsellor asked 33 questions, whereas the GPs asked only 2 questions. After identifying and counting the speech functions of checking, confirming, clarifying, disengaging, challenging, and countering, it was found that the counsellor used these speech functions approximately three times more often than the GPs did, i.e., 86 times versus 27 times. This comparison signifies that the counsellor proactively used different speech functions to clarify the information being exchanged, and to ensure the preciseness of the information they acquired.

**Table 2:** Speech functions in GP–counsellor interactions.

	GPs	Counsellor
Statements	505	449
Yes/No Questions	2	22
Wh-Questions	0	11
Acknowledgements	163	236
Checking	1	10
Confirming	12	25
Clarifying	6	23
Disengaging	1	0
Challenging	1	8
Countering	6	20

The counsellor’s high frequency in using a wide range of speech functions may signal the most important kinds of knowledge that the GPs and counsellor were exchanging in their case meetings. In addition, the reason why the counsellor used many of the speech functions far more often than the GPs may shed light on their differing understandings of their professional roles, along with their different ideologies and modalities. As has been mentioned previously, GPs and counsellors tend to perceive and deal with patients’ mental health issues from different epistemological and ontological perspectives. Understanding these differences may help enhance IPC through more effective communication. To illustrate how the GPs and the counsellor communicated by using a range of speech functions, the following sections focus on the interactional sequences.

A new patient, KM, was a 40-year-old married woman living with her husband. She had been consulting a psychiatrist for anxiety and depression for over 10 years, first in a public hospital, and then in a private setting. She had grown frustrated by reoccurring anxiety symptoms and incessant medications. In searching for a possible way out, she finally came to see if the GPs at the private clinic could help (Excerpt 1).

#### Excerpt 1

**Table:** 

Exchange	Speaker	Talk	Speech Function(s)
1	GP:	She has been seeing the psychiatrist for eight, ten years.	
2	C:	A private psychiatrist?	**Clarifying**
3	GP:	…those who wanted to help her prescribed a heavy dosage of drugs with also benzodiazepine. Told her that she needed to take if for the whole life. She doubted if it’s true. After a few years of taking benzodiazepine, she was unable to stop it. She treated it like jewellery.	
4	C:	Keep taking the benzodiazepine!	**Confirming**
5	GP:	…she did not want to lose. She surfed on the Internet and got us somehow. She came to see me finally, and today is her second consultation.	
6	C:	Seen you twice in total.	**Confirming**

In exchange 1, the GP and the counsellor discussed KM’s case by exploring her previous clinical encounters, and used a range of interactional sequences realized by various speech functions (e.g., clarifying, confirming). The GP contributed a significant amount of talk by making statements to give his interpretations of the condition, and the counsellor responded with acknowledgements. The counsellor also deliberately used many clarifications to confirm the information about medications that the GP had prescribed, and to prompt more responses from him.

In exchange 2, when the GP told the counsellor that KM had seen psychiatrists for over 10 years, the counsellor immediately intervened and asked for clarification on whether the psychiatrist was in private practice or under the Hospital Authority. As the counsellor, he was considering the extent of the quality time and support that a psychiatrist in the Hospital Authority would be able to provide for the patient. In exchange 4, when the GP mentioned the drug ‘benzodiazepine’, a commonly prescribed tranquilizer, the counsellor immediately sought confirmation of whether KM had been taking this drug for a long time. In fact, this information aroused the counsellor’s concern because many patients with anxiety problems easily develop a dependence on tranquilizers. In exchange 6, the counsellor again used the confirmation strategy. He sought the GP’s confirmation of whether KM had come to the clinic only twice. This information let the counsellor assesses the degree of rapport between the GP and KM. From the perspective of the counsellor, KM’s degree of trust in the GP could have a crucial impact on her decision-making, for instance on whether she would adopt a new treatment plan and consult a new counsellor. The six turns explicitly encompass the themes of the patient’s previous clinical encounters, her presenting problems, medication use and management, her expectations, and her level of rapport with GP. These interactional sequences reveal the kinds of information being exchanged between the GP and the counsellor.

#### Excerpt 2

**Table:** 

Exchange	Speaker	Talk	Speech Function(s)
1	GP:	I think her condition is not so bad. She actually tailed down the drugs. So why not just keep it. It seems that taking the anti-depressant alone helps tail off the benzodiazepine successfully.	
2	C:	Did she tail down the drugs deliberately?	Checking
3	GP:	Sure, she did it intentionally. By the way, there’s something wrong with her dad. She said that she was sexually abused by her dad when she’s small. Her mum did not believe her. The story is kind of the typical ones. Recently, she has not seen her dad for a month. Cut down the contact. A whole month already. Then she felt more comfortable.	Confirming
4	C:	Did she mention why she chose not to see her dad at this particular time?	Clarifying
5	GP:	Because she felt really struggling!	Disengaging
6	C:	But why this particular reaction at this particular moment?	Challenging
7	GP:	Right. I think it is partially due to her husband. He made this suggestion. Her husband is somehow supportive, coming with her. It seems to me that her husband has been supporting her to see the doctors over the years. But she has stopped working since 2009, the onset of her mental illness.	Acknowledgement

In Excerpt 2, the GP continued with the recommendation that KM should tail off using the benzodiazepines on her own. The counsellor then moved to check on this recommendation by asking a closed-ended question, ‘Did she tail down the drugs deliberately?’ Tailing down the drugs deliberately could signify her motivation and determination to change. This information was important for the counsellor, which is then confirmed by the GP.

In exchange 3, the GP informed the counsellor that KM had disclosed her experience of childhood sexual abuse by her father, and that she had ‘cut down the contact’ with him for a month. The counsellor then moved to clarify the reason for this change in her behavior. In exchange 5, the GP seemed to regard the reason as obvious, and moved to terminate the interaction by saying, ‘Because she was really struggling!’ However, the counsellor returned to this question, asking the reason for KM’s sudden change at that particular moment. The GP’s response signified his acceptance of the counsellor’s challenge. He was then prompted to give additional information acquired from his clinical observations.

The above-described interactional sequences again revealed the different salient strategies that the counsellor adopted to check, confirm, and clarify the GP’s statements. These moves revealed the essential information being exchanged, such as the patient’s significant emotional response that could have signaled her childhood sexual abuse experience. By using the speech function of challenging, the counsellor successfully increased the GP’s awareness of the possible causes behind KM’s subtle change of behavior. Although the real cause remained unknown, this incident provided an important entry point for exploring KM’s psychological condition.

#### Excerpt 3

**Table:** 

Exchange	Speaker	Talk	Speech Function
1	GP:	I have explained it to her today. I told her it may sound like a wardrobe. You may not necessarily open all the doors. You may just want to choose the one for jackets. She has probably seen social work trained counsellors before. They might handle her issue of sexual abuse too early. Since there are so many problems to deal with, we may start with something superficial.	
2	C:	Or looking for her strengths and resources first. That is fundamental.	Countering
3	GP:	Yes, certainly!	Acknowledgement
4	C:	It’s too scary! She has already suffered from so many somatic symptoms.	Confirming

In Excerpt 3, the GP talked about taking another perspective and starting with more superficial issues. In exchange 2, the counsellor responded to this strategy by saying, ‘Or looking for strengths and resources first. This is fundamental.’ He moved to offer an alternative perspective on therapy. In exchange 3, the GP expressed his acknowledgement by agreeing completely with the counsellor’s viewpoint. Following the GP’s acknowledgement, the counsellor even confirmed the GP’s viewpoint by providing a reason for his advice given in exchange 4, “It’s too scary! She has already suffered from so many somatic symptoms.”

In summary, the counsellor deliberately crosschecked the patient’s information with the GP for two explicit communicative purposes. The first purpose was to ensure the preciseness of the information provided. The second was to seek additional information on the GPs major concerns, for instance the patient’s previous clinical encounters, the management of medication, the patient’s expectations, and the patient’s significant emotional events.

**Figure 1:**
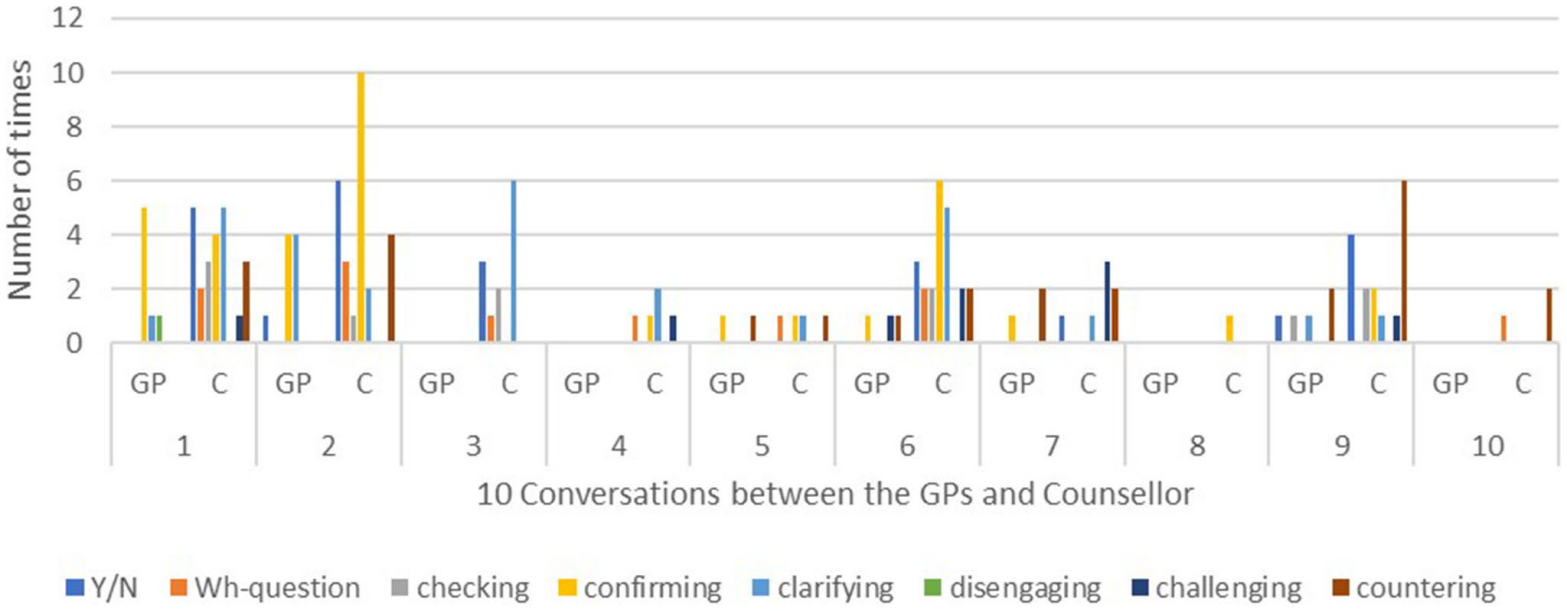
Use of speech functions in 10 conversations between the GPs and the counsellor.

These interactional strategies constructed the sequences of the talk and shaped the interaction surrounding the essential themes that reflected the primary concerns of the GP, or the counsellor, or both, in therapy and in their collaborative partnership. Reducing symptoms and balancing neurotransmitters in the brain are often the immediate concern of GPs, who are trained in the biomedical model. In contrast, counsellors generally perceive mental distress from a wider perspective, i.e., the bio-psycho-social-spiritual model. In addition to considering biochemicals and brain functions, they may ask many questions about interpersonal and intrapersonal relationships, emotional episodes, resiliency, and values and beliefs. This broader perspective in part explains why the counsellor used far more speech functions in the conversations than the GPs, as shown in Figure 1.

### Topics in the GPs’ and counsellor’s conversations

The contents of these conversations can be summarized in 10 main topics: (1) medication management; (2) case management; (3) the patient’s previous clinical encounters; (4) the patient’s presenting problems; (5) the patient’s expectations; (6) rapport building; (7) the GPs’ conceptualizations of the cases; (8) counsellor’s case conceptualization; (9) the patients’ significant emotional events and (10) clinical observation of each patient’s current condition. These reoccurring topics signified the major concerns of the GPs and the counsellor. The first two topics were the most prominent concerns, and emerged in every single conversation. In addition to the above-mentioned topics, the analysis of the counsellor’s back-stage behaviors, as observed in his self-reflection logs, uncovered five remarkable themes. The following section reports the reflections of the counsellor.

#### Medication management

The theme of medication management was the crux of the GP-counsellor interaction. In Excerpt 4, the patient MT was in his mid-thirties. He had been diagnosed with bipolar affective disorder, and was on medication.

##### Excerpt 4

**Table:** 

C:	… But I suppose he will say that again. Shall I not take the medicine? I believe he will say that again!
GP:	Treat MT as a child, and it should be alright!
GP:	Alright.
C:	Sweet-talk, then persist, sweet-talk, then persist, that will be alright!

In this case meeting, the counsellor told the GP that the patient sought to stop medication and wanted to know how the GP would respond to that. The GP responded by encouraging the counsellor to persuade the patient to continue the medication. However, the counsellor was also struggling inside, because he understood why the patient wanted to stop or reduce the medication. Over the past 6 months or more, the patient’s daily functioning, such as his sleep, appetite, and work competence, had returned to normal. To a large degree, the symptoms were being well-managed. The only problem was that the side effects of the medication always made MT feel lethargic. He was very frustrated by his poor quality of life.

In this case, the counsellor knew ‘privately’ that the GP might not be willing to risk the experiment of reducing the medication, because the constant use of mood stabilizers was considered crucial to the treatment of bipolar affective disorder. The GP might consider reducing the treatment to be risky. The counsellor responded without hesitation by saying ‘alright!’ to maintain his ‘professional self’ and show understanding regarding the importance of using mood stabilizers, and to avoid embarrassing the GP. However, deep down in the counsellor’s mind, he wanted the GP to contribute to the experiment in overcoming drug dependence. It will be very encouraging if the GP could spend more time to listen to and understand the patient’s daily life struggles empathetically. The patient, the GP, and the counsellor may work as a team on making a shared decision.

In general, eliminating symptoms is the primary concern of medical doctors, whereas counsellors read important messages about the patients from their symptoms. Counsellors often view symptoms as signals, which help them investigate the root causes of the patient’s mental distress. Medication is sometimes understood as an intrusive way of fixing symptoms and eliminating the distress. In the bio-medical paradigm, the symptoms are viewed as the cause of distress, rather than signals that indicate the real problem. In fact, both GPs and counsellors sometimes face dilemmas over why, when, and how to use or cease using certain medications. Nonetheless, we believe that with more effective communication between GPs and counsellors, a better balance can be found between the physical, psychological, social, and quality of life considerations for the patients’ overall welfare.

#### Counsellor’s case conceptualization

In analyzing the conversational data, we found that the counsellor had at least two major concerns in assessing the new patients’ conditions, which differed from the GP’s priorities. The first was about the patients’ significant others, such as spouses or parents, and the second was the patients’ readiness for entering counselling.

The Excerpt 5 illustrates one example in a case meeting where the counsellor found himself very eager to explore the patient’s relationship with his wife, because the patient’s infertility problem was presented as his major source of emotional distress. This, however, was not the GP’s primary concern in his first encounter with the patient.

##### Excerpt 5

**Table:** 

C:	… I would like to see if he has mentioned anything about the relationship with his wife during this process.
GP:	Um… not much.
C:	Not much!
GP:	Ye…s… wife… not mention too much, about that.
C:	Because he was talking about infertility. I wonder. How is he with his wife? It is not just his business!
GP:	He has not mentioned it.

In addition, the patient’s readiness for entering counselling is an important concern for a counsellor as he or she prepares for the first encounter with a patient. Instead of being something that miraculously or suddenly occurs, this kind of readiness is always a gradual process that unfolds over time. Recovery is fundamentally about the motivation to change. Therefore, how ready the patient is to change, in terms of making the decision to enter counselling, is a critical factor in the effectiveness of therapy (Prochaska et al., 1994).

According to the counsellor’s observations, ‘doing counselling’ was sometimes the preference of the GP rather than that of the patient, even though it was critical to introduce the patient to counselling as a choice (Lambert, 2013). The counsellor was therefore very much concerned about the process of shaping the patient’s decision-making. We observed that the counsellor frequently checked with the GP about whether the patients were ready to do counselling, and about how the patients finally came to their decisions. The counsellor perhaps experienced this concern during IPC communication, knowing that the GP could override the decision-making process.

#### Case management

It is interesting to consider the GPs and the counsellor’s possibly divergent evaluations of treatment effectiveness. Their front-stage behaviors regarding the treatment goals for the patients seemed to be the same. However, their concerns over the effectiveness of treatments could differ. Excerpt 6 reveals a situation in which the GP and the counsellor were considering and articulating different interpretations about the therapeutic effectiveness. The patient was suffering from severe post-traumatic symptoms due to the physical and emotional violence imposed by her husband. In the meantime, she was suffering from bipolar affective disorder and experienced episodes of elation followed by depression. The treatment goal or concern that was agreed between the GP and the counsellor was the management of symptoms, because the symptoms greatly impaired her daily functioning.

##### Excerpt 6

**Table:** 

GP:	… not very stable indeed. What I mean is that she is not very depressed over things frustrating her. She is also not very irritable over things irritating her.
C:	Yep.
GP:	So I think her condition is acceptable.
C:	But I suppose the greatest improvement is the fading of traumatic symptoms. All gone.

This patient first appeared to present severe post-traumatic stress symptoms due to domestic violence and it was an extremely difficult period of her life. The counsellor can still recall her trembling body, shaking voice, flashbacks, self-defeating thoughts, and chronic depressive symptoms. The patient had been receiving joint care from the GP and the counsellor for 3 years or more, twice a month. By the point of the recorded discussion, her physical and mental conditions were good enough for working and socializing normally, and her vitality had revived.

The counsellor had been working hard to help her deal with her trauma. He perceived her traumatic experiences as very significant emotional events that had affected most areas of her life, for instance in terms of poor sleep, appetite, troubled thinking and memory, and impaired social competence. Therefore, the counsellor’s ultimate concern was how she perceived and responded to her past, present, and future. The GP, however, considered her mood fluctuations as the index of her mental wellness. They were working on the same problem and its solution on the front stage, but interpreted the outcome of therapy from different points of view on the back-stage. These differing goals imply their different focuses of concern. Their epistemological and ontological perspectives regarding mental health and wellness were divergent. From the GP’s perspective, the elimination or reduction of symptoms ultimately signified the patient’s recovery from mental distress. In contrast, the counsellor’s major concern was always about the patient’s new perspective, the narrative of her past traumatic experience and her moment-to-moment daily experience.

#### Knowledge transfer

IPC facilitates knowledge transfer between different disciplines. When the GP and the counsellor were discussing cases, they acquired new knowledge from the other party’s professional discourse and discipline. Their decisions were the by-products of collaborative partnership. In 4 out of the 10 conversations, there were instances where the GP and the counsellor unexpectedly exchanged the terminologies of their disciplines. In the Excerpt 7, the counsellor introduced the terms of core emotions and emotional flashback to the GP. The Excerpt 7 describes one of these scenarios.

##### Excerpt 7

**Table:** 

C:	Just like this.
GP:	Should be ok. I mean o, o, o, okay! You are okay too! What I mean is like I see the whole picture. When you are doing a surgery, I’ll wrap things up beside you! You’re doing something sophisticated!
C:	Good. And there lays the support for her by the medication.
GP:	Yes. To work collaboratively like this sounds good!
Cw:	Yes. Thank you!

In this Excerpt 7, it was observed that the GP and the counsellor were really working as a team with an explicit division of work, which was articulated with a metaphor adopted by the GP. As an insider in this working relationship, the counsellor asserted that the acknowledgement of each other’s expertise was crucial to maintaining and fostering the effectiveness of their collaboration, which in fact offered benefits to both the health care providers and their patients. Bosch and Mansell (2015) confirmed this notion that patient outcomes have been improved by collaboration in health care. The improvements they observed included reductions in preventable adverse drug reactions, declines in morbidity and mortality rates and optimization of medication dosages. Teamwork has also been shown to provide benefits to health care providers, including reductions of extra work and increased job satisfaction.

#### Acknowledging the partnership

Situating in a private clinic, the counsellor offers mental health counselling to the patients referred by the GPs. Together, they discuss and work on the cases aiming to provide the patients with a holistic care approach to disease. In this context, they are motivated to maintain and foster the collaborative partnership by desired outcomes. One of the desired outcomes is certainly the patients’ wellness. On top of it, the counsellor wrote in the reflection log another critical factor from the conversations which may foster the GP-counsellor collaboration. This is the acknowledgement of each other’s expertise (Excerpt 8).

##### Excerpt 8

**Table:** 

GP:	Just like this.
C:	Should be ok. I mean o, o, o, okay! You are okay too! What I mean is like I see the whole picture. When you are doing a surgery, I’ll wrap things up beside you! You’re doing something sophisticated!
GP:	Good. And there lays the support for her by the medication.
C:	Yes. To work collaboratively like this sounds good!
C:	Yes. Thank you!

From this conversation, it was observed that the GP and the counsellor were really working as a team with an explicit division of work, articulated with a metaphor adopted by the GP. As an insider in this working relationship, the counsellor opines that the acknowledgement of each other’s expertise is crucial to maintain and foster the effectiveness of this collaboration which in fact offers benefits to both health care providers and patients.

## Discussion

By using the ethnographic discourse analysis approach, this study unpacked the nature of GP-counsellor interactions during IPC on three levels: (a) structural mapping (i.e., counting the verbal contributions in discussions between the GPs and the counsellor); (b) thematic mapping (i.e., identifying the recurring themes discussed by the GPs and the counsellor) and (c) discourse mapping (i.e., the interactional strategies used by the GPs and the counsellor for clarifying their understandings regarding patients’ conditions). The data showed that the GPs and the counsellor mainly discussed their patients’ issues in terms of medication management, the counsellor’s case conceptualization, the case management, knowledge transfer and acknowledging the partnership. The knowledge transfer and acknowledging the partnership were the least recurrent themes in the discussions between the GPs and the counsellor. During case discussions, both the GPs and the counsellor used a range of interactional strategies to clarify a patient’s condition and treatment plans for mutual understanding.

Five major themes were generated in the analysis of the participants’ reflections. Three of these themes overlapped with the themes derived from the thematic mapping analysis, namely medication management, counsellor’s case conceptualization and case management. The interconnections between these themes further confirmed their validity. These findings supplemented the answers to the research questions about the information being exchanged in the GP-counsellor interactions, and about the ways these partners communicated. Excluding the 10 reoccurring topics and the overlapping themes identified in the data, there were two particular themes derived from the reflections on the counsellor’s back-stage behavior, namely knowledge transfer and acknowledging the partnership. The findings on these two themes can be considered fully applicable to all kinds of IPC.

Furthermore, the counsellor’s reflective accounts offer great insights into the professional experience of IPC. Generally, doctors and other medical staff members step into their professional characters and deliver specialized medical discourses during clinical encounters with patients/clients. In GP-counsellor interactions, however, they talk as co-workers instead of as doctors treating their patients. The co-workers co-construct an insider discourse that covers a greater diversity of topics, including both medical and non-medical concerns (Barton, 2004).

When professionals communicate with the patients and their next of kin, they might offer hope, but this should be carefully balanced with realism (Barton, 2004). According to Barton (2004), the job of a medical oncologist is to balance realism and hope. As noted previously, there is no health without mental health (World Health Organization and World Organization of Family Doctors, 2008), and it is commonly suggested that mental disorders are among the most significant causes of death across the world (Walker et al., 2015). Many severe mental issues such as depression are often fatal, but are still commonly ignored. These illnesses should be given more attention, and they should be treated as seriously as other commonly lethal illnesses such as cancer (Ledford, 2014).

In Barton (2004)‘s study regarding clinical discourses on oncology, it was argued that paying close attention to the discourse on medical prognosis during IPC communication could help indicate whether there was a need for balancing between realism and hope. Clinical professionals may make backstage comments about cases, especially when the cases are worrisome (Barton, 2004). By staying alert during their back-stage discourses, professionals may be enabled to re-examine their conversations with the patients/clients and families. They may discern ways to avoid false hope, and to ensure that everything provided for the patients/clients is sufficient and correct. It is often presumed that decisions are made during interactions between provider and patient, but decision-making activity also occurs in IPC communication (Coombs, 2004). Therefore, professionals, GPs and counsellors might consider making decisions through reviewing their IPC discourse alongside their formal encounters with patients (Ellingson, 2003).

As with all research, our data are presented alongside their limitations. The current research is a small-scale study of GP-counsellor communication. Since the results are based on a single clinic and a small sample size (i.e., three participants), they are limited in generalizability. However, although the participants’ experiences may not necessarily reflect those of healthcare professionals worldwide, they are highly relevant to healthcare professionals whose daily work involves IPC.

With regard to theory implications, we gave Pun et al. (2017)‘s ethnographic discourse analytical approach an empirical drive in IPC. It is a useful heuristic for a discourse analytic account of communication processes in healthcare contexts and, as this study suggests, of how analysis of IPC communication should extend beyond the focus of information exchange (Eisenberg, 2008). As such, this approach helpfully illustrates the medical and interpersonal aspects of IPC, notably by accounting for how GPs and counsellors help each other understand the complexities of the patient’s condition through bringing to bear their professional knowledge, expertise, and interpretations. The identified discourse features of GP-counsellor interactions provide a possible framework for developing more effective communication, with a view to enabling insight regarding the primary concerns for a better understanding of patients’ mental health issues.

## Conclusion

GPs are primarily concerned about making diagnoses, the use of medications and the elimination of symptoms. The comparatively high frequency with which the counsellor in this study used speech functions for seeking clarification and additional information implies that the counselling modality is focused on many aspects of a patient’s mental health issues beyond the biochemical factors. For instance, the counsellor raised concerns about personality traits, interpersonal and intrapersonal relationships, stressors, coping stances, insights, values, beliefs, and other factors. The identification of the key kinds of information or themes exchanged between the GPs and the counsellor offers a possible framework for developing effective communication between GPs and counsellors. This key information also reflected the primary concerns of the GPs and the counsellor for understanding the patients’ mental health issues. This proposed framework can be adopted for education programs, as training for the interdisciplinary collaboration of GPs and counsellors.

This study is preliminary and exploratory in nature. The results therefore provide a point of entry for more research studies regarding professionalized discourse in GP-counsellor communication. The key kinds of information discussed and the interactional strategies applied in communication between GPs and counsellors are seen as critical elements for developing a comprehensive framework for pedagogical content. Future research may seek to develop this study on a larger scale and how cultural values may impact the interaction between GP and counsellor.

## Data availability statement

The original contributions presented in the study are included in the article/supplementary material; further inquiries can be directed to the corresponding author.

## Ethics statement

The studies involving human participants were reviewed and approved by the CLASS human research ethics committee of the City University of Hong Kong. The patients/participants provided their written informed consent to participate in this study.

## Author contributions

All authors listed have made a substantial, direct, and intellectual contribution to the work and approved it for publication.

## Conflict of interest

The authors declare that the research was conducted in the absence of any commercial or financial relationships that could be construed as a potential conflict of interest.

## Publisher’s note

All claims expressed in this article are solely those of the authors and do not necessarily represent those of their affiliated organizations, or those of the publisher, the editors and the reviewers. Any product that may be evaluated in this article, or claim that may be made by its manufacturer, is not guaranteed or endorsed by the publisher.
